# Supplementary material for: Comparing the safety and efficacy of sodium valproate, levetiracetam, and phenytoin in attenuating the severity of agitation in patients with post-traumatic brain injury: An observational study
Source: PLoS One. 2026 Jun 3;21(6):e0350585. doi: 10.1371/journal.pone.0350585 (PMC13232942; doi:10.1371/journal.pone.0350585)
Supplement: Attachment — Submitted filename: Response to Reviewers.docx [file pone.0350585.s001.docx]

1. Please ensure that your manuscript meets PLOS ONE's style requirements, including those for file naming. The PLOS ONE style templates can be found at

[https://journals.plos.org/plosone/s/file?id=wjVg/PLOSOne_formatting_sample_main_body.pdf](https://track.editorialmanager.com/CL0/https:%2F%2Fjournals.plos.org%2Fplosone%2Fs%2Ffile%3Fid=wjVg%2FPLOSOne_formatting_sample_main_body.pdf/1/010f019cb2ecadb0-27a783c9-34ac-4c08-9414-c11de1132f86-000000/696PnSFlb7cE8MdfD3qXmSDcF8ONMAPlD0dFLcZsuYw=252) and [https://journals.plos.org/plosone/s/file?id=ba62/PLOSOne_formatting_sample_title_authors_affiliations.pdf](https://track.editorialmanager.com/CL0/https:%2F%2Fjournals.plos.org%2Fplosone%2Fs%2Ffile%3Fid=ba62%2FPLOSOne_formatting_sample_title_authors_affiliations.pdf/1/010f019cb2ecadb0-27a783c9-34ac-4c08-9414-c11de1132f86-000000/sU4ZRUfsgQaMKXJuC-2c5-Iqc2KNNzxe-vkZ8QNvSAE=252)

**Response:** Thank you sir. We have complied with the journal style

2. Thank you for stating the following in your Competing Interests section:

[NO authors have competing interests].

Please complete your Competing Interests on the **online submission form** to state any Competing Interests. If you have no competing interests, please state "The authors have declared that no competing interests exist.", as detailed online in our guide for authors at <http://journals.plos.org/plosone/s/submit-now>

This information should be included in your cover letter; we will change the online submission form on your behalf.

**Response**: We have stated "The authors have declared that no competing interests exist" and the same is included in the Cover letter.

3. We note that your Data Availability Statement is currently as follows: [All relevant data are within the manuscript and its Supporting Information files.]

Please confirm at this time whether or not your submission contains all raw data required to replicate the results of your study. Authors must share the “minimal data set” for their submission. PLOS defines the minimal data set to consist of the data required to replicate all study findings reported in the article, as well as related metadata and methods (https://journals.plos.org/plosone/s/data-availability#loc-minimal-data-set-definition).

For example, authors should submit the following data:

- The values behind the means, standard deviations and other measures reported;
- The values used to build graphs;
- The points extracted from images for analysis.

If your submission does not contain these data, please either upload them as Supporting Information files or deposit them to a stable, public repository and provide us with the relevant URLs, DOIs, or accession numbers. For a list of recommended repositories, please see <https://journals.plos.org/plosone/s/recommended-repositories>.

**Response**: Dear Sir, we have provided the compiled raw dataset used in the study as a supporting information file for your reference along with codes [S1_Rawdata and S2_Diary].

4. When completing the data availability statement of the submission form, you indicated that you will make your data available on acceptance. We strongly recommend all authors decide on a data sharing plan before acceptance, as the process can be lengthy and hold up publication timelines. Please note that, though access restrictions are acceptable now, your entire data will need to be made freely accessible if your manuscript is accepted for publication. This policy applies to all data except where public deposition would breach compliance with the protocol approved by your research ethics board. If you are unable to adhere to our open data policy, please kindly revise your statement to explain your reasoning and we will seek the editor's input on an exemption. Please be assured that, once you have provided your new statement, the assessment of your exemption will not hold up the peer review process.

**Response**: Thank you for the clarification. We confirm that the submission contains all data required to replicate the study findings, including the values underlying summary statistics, figures, and analyses, which are provided as Supporting Information.

The dataset has been fully anonymized and includes all variables necessary for reproducibility. Although informed consent was obtained, explicit consent for unrestricted public data sharing was not secured at the time of enrolment from the participants. We therefore consulted the Institutional Ethics Committee, and in accordance with their guidance, full open access to the dataset is restricted to ensure participant confidentiality. The raw data are available and maintained within the Institutional Ethics Committee repository; access will be provided upon request. Requests for access can be directed to [**iec.kmc@manipal.edu**](mailto:iec.kmc@manipal.edu) to ensure compliance with ethical and legal requirements.

5. PLOS requires an ORCID iD for the corresponding author in Editorial Manager on papers submitted after December 6th, 2016. Please ensure that you have an ORCID iD and that it is validated in Editorial Manager. To do this, go to ‘Update my Information’ (in the upper left-hand corner of the main menu), and click on the Fetch/Validate link next to the ORCID field. This will take you to the ORCID site and allow you to create a new iD or authenticate a pre-existing iD in Editorial Manager.

**Response**: Dear Sir, the orcid ID of the corresponding author is validated in Editorial Manager. Corresonding author’s ORCID ID is [0000-0002-6359-4024](https://www.scopus.com/redirect.uri?url=https://orcid.org/0000-0002-6359-4024&authorId=24503464400&origin=AuthorProfile&orcId=0000-0002-6359-4024&category=orcidLink)

6. We note that Figure 4 in your submission contains images which may be copyrighted. All PLOS content is published under the Creative Commons Attribution License (CC BY 4.0), which means that the manuscript, images, and Supporting Information files will be freely available online, and any third party is permitted to access, download, copy, distribute, and use these materials in any way, even commercially, with proper attribution. For more information, see our copyright guidelines: <http://journals.plos.org/plosone/s/licenses-and-copyright>.

**Response**: Sir, we thank you for highlighting this important point. Figure 4 was created by myself (Dr Shivaprakash G and Dr Shivani) using BioRender under an institutional academic license.

We have obtained and uploaded the official BioRender “Confirmation of Publication and Licensing Rights – Open Access” document, which explicitly permits publication of the figure under a CC BY 4.0 license, including for journals such as Public Library of Science.

We confirm here that the figure does not contain any third-party copyrighted material. In accordance with BioRender’s licensing requirements, the appropriate citation has been included in the revised figure caption. [*Created in BioRender. Parida, A. (2026) https://BioRender.com/orogd49*]

We confirm that the figure fully complies with the journal’s copyright and licensing requirements.

**Reviewers' comments:**

Reviewer's Responses to Questions

**Comments to the Author**

1. Is the manuscript technically sound, and do the data support the conclusions?

The manuscript must describe a technically sound piece of scientific research with data that supports the conclusions. Experiments must have been conducted rigorously, with appropriate controls, replication, and sample sizes. The conclusions must be drawn appropriately based on the data presented.

Reviewer #1: Yes

**Response**: Thank you Sir.

2. Has the statistical analysis been performed appropriately and rigorously?

Reviewer #1: No.

**Response**: Dear Sir, we thank the reviewer for this important comment. The entire dataset was reanalyzed in consultation with an independent statistician, with analyses performed using the latest version of R software and appropriate statistical methods. All results were cross-verified to ensure accuracy and consistency. The detailed statistical methodology has been clarified in the revised “Statistical Analysis” section, and the raw dataset has been provided for transparency. We confirm that the analyses performed are appropriate and robust for the study and can be independently verified.

3. Have the authors made all data underlying the findings in their manuscript fully available?

The [PLOS Data policy](http://track.editorialmanager.com/CL0/http:%2F%2Fwww.plosone.org%2Fstatic%2Fpolicies.action%23sharing/1/010f019cb2ecadb0-27a783c9-34ac-4c08-9414-c11de1132f86-000000/obRW7dgTLCuabmSyeCZHqISBCDNBO64IXdXEeF-wZgw=252) requires authors to make all data underlying the findings described in their manuscript fully available without restriction, with rare exception (please refer to the Data Availability Statement in the manuscript PDF file). The data should be provided as part of the manuscript or its supporting information, or deposited to a public repository. For example, in addition to summary statistics, the data points behind means, medians and variance measures should be available. If there are restrictions on publicly sharing data—e.g. participant privacy or use of data from a third party—those must be specified.

Reviewer #1: Yes

**Response**: Thank you Sir.

4. Is the manuscript presented in an intelligible fashion and written in standard English?

PLOS ONE does not copyedit accepted manuscripts, so the language in submitted articles must be clear, correct, and unambiguous. Any typographical or grammatical errors should be corrected at revision, so please note any specific errors here.

Reviewer #1: Yes

**Response**: Thank you Sir.

5. Review Comments to the Author

Please use the space provided to explain your answers to the questions above. You may also include additional comments for the author, including concerns about dual publication, research ethics, or publication ethics. (Please upload your review as an attachment if it exceeds 20,000 characters)

Reviewer #1: The authors of PONE-D-25-68208 conducted a prospective study to compare the safety and efficacy of sodium valproate, levetiracetam, and phenytoin in agitation control in patients managed after traumatic brain injury.
The study concludes that sodium valproate has higher efficacy and rapid beneficial actions in reducing agitation compared to levetiracetam, and the authors suggest that this treatment strategy offers better safety profile than phenytoin and levetiracetam.

I believe that the study was well designed and reported, the reference list is extremely poor and would benefit from inclusion of other relevant works. I do not totally agree with the conclusion that sodium valproate is easier to use than levetiracetam and would also modify the conclusion accordingly. Please see my detailed points below:

The introduction would benefit from additional references on delirium incidence, pathophysiology and management in NeuroIntensive Care Unit and in particularly following admission of silver traumas, given the increased risk in elderly patients. Please consider the followings:
- O'Keeffe F, et al. Serum biomarkers of delirium in critical illness: a systematic review of mechanistic and diagnostic evidence. Intensive Care Med Exp. 2025;13(1):90. doi: 10.1186/s40635-025-00795-z.
- Depreitere B, et al. Unique considerations in the assessment and management of traumatic brain injury in older adults. Lancet Neurol. 2025;24(2):152-165. doi: 10.1016/S1474-4422(24)00454-X.

I would also suggest to provide a better rationale for the use of antiepileptics, the authors provide only one paragraph about that. Ganau et al. provided a clear rationale for such pharmacological class and I would suggest to refer to their section dedicated to antiepileptics:
- Ganau M, et al. Delirium and agitation in traumatic brain injury patients: an update on pathological hypotheses and treatment options. Minerva Anestesiol. 2018;84(5):632-640. doi: 10.23736/S0375-9393.18.12294-2.

The antiepileptic drugs suggested are very effective in TBI patients however at present there is no equipoise on the most appropriate strategy, in fact the randomized trial MAST (see <https://clinicaltrials.gov/study/NCT04573803>) is still enrolling patients to clarify this matter. At present the only consensus is on the use of sedation plus poly-pharmacological strategy for the management of status epileptics following TBI (see Prisco L, et al. A pragmatic approach to intravenous anaesthetics and electroencephalographic endpoints for the treatment of refractory and super-refractory status epilepticus in critical care. Seizure. 2020;75:153-164. doi: 10.1016/j.seizure.2019.09.011.).
A consideration on those important pieces of scientific research is certainly warranted.

**Response**: We sincerely thank the reviewer for the constructive feedback and for highlighting relevant literature to strengthen the manuscript.

As suggested, we have substantially revised the Introduction to improve the depth and context of the study. Specifically, we have incorporated additional references addressing the incidence, pathophysiology, and management of delirium in neurocritical care settings, with particular emphasis on older adults and trauma populations.

- Burden: Lines 120-127
- Pathophysiology: Lines 128-136
- Management: Lines 137-165

We have also expanded the rationale for the use of antiepileptic drugs in post-traumatic agitation providing a clearer mechanistic and clinical basis for this pharmacological approach.

- Rationale of using antiepileptics: Lines 167-181

We have revised the conclusion to avoid overstating the comparative ease of use of sodium valproate; the final conclusion is now data-driven and has been modified in accordance with the available evidence.

Conclusion: Lines 395-399

We believe these changes have significantly improved the scientific rigor and contextual relevance of the manuscript and hope will satisfy, point by point to reviewers’ queries.
